# Assessment of job satisfaction, self-efficacy, and the level of professional burnout of primary and secondary school teachers in Poland during the COVID-19 pandemic

**DOI:** 10.7717/peerj.13349

**Published:** 2022-06-10

**Authors:** Anna Bartosiewicz, Edyta Łuszczki, Lech Zaręba, Maciej Kuchciak, Gabriel Bobula, Katarzyna Dereń, Paweł Król

**Affiliations:** 1 Institute of Health Sciences, Medical College of Rzeszów University, Rzeszów, Poland; 2 Institute of Computer Science, College of Natural Sciences, University of Rzeszów, Rzeszów, Poland; 3 Institute of Physical Culture Sciences, Medical College of Rzeszów University, Rzeszów, Poland

**Keywords:** Burnout, COVID-19, Job satisfaction, Self-efficacy, Teachers

## Abstract

**Background:**

The work of teachers during the COVID-19 pandemic created additional challenges and required them to go beyond conventional teaching methods, which in turn required teachers to be more resilient and caused stress and excessive workload. The aim of the study was to assess the level of occupational burnout, the sense of job satisfaction and the self-efficacy of primary and secondary school teachers working during the COVID-19 pandemic in Poland.

**Methods:**

This is a cross-sectional descriptive study conducted among 412 teachers from randomly selected primary and secondary schools in the Podkarpacie region in Poland. The questionnaire contained socio-demographic data of the respondents and three standardized scales: The Scale of Job Satisfaction (SJS), General Self-Efficacy Scale (GSES) and Arbeitsbesorgenes Verhaltens und Erlebenmuster (AVEM). The Shapiro–Wilk, Kruskal–Wallis and Mann–Whitney *U*-test were used. Statistical analysis was performed with Statistica TIBCO 13.3 and R (version 3.6.1) software.

**Results:**

The satisfaction with the work of the surveyed teachers was average in the job satisfaction scale. The vast majority of the surveyed teachers presented type *B (*overburdened risk type) of work-related behavior, and type *A* (overburdened risk type) in which the probability of burnout is very high. The feeling of self-effectiveness determines the level of job satisfaction and the level of professional burnout among the surveyed teachers. Job satisfaction had a statistically significant impact on the assessment of self-efficacy of the surveyed teachers (*p* < 0.0001). The types of work-related behavior have a statistically significant impact on the assessment of the teachers’ own effectiveness (*p* < 0.0001).

**Conclusion:**

There is a close relationship between teachers’ level of self- efficacy, job satisfaction and, therefore it may predispose them to the occurrence of burnout syndrome in the future. Support for this professional group is needed. These findings may be important for education policy, teacher’s practice, and subsequent research. Ultimately, the study may provide some suggestions for methodological and educational strategies.

## Introduction

Since March 2020, full-time education has been resumed at the beginning of the 2020/2021 school year for a period of one and a half months. Children in grades 1–3 were temporarily returned to school, but in total it was not longer than 2 months. The gradual return of all primary and secondary school students began in mid-May 2021, *i.e.,* after more than a year of physical absence of children and adolescents in educational institutions. However, Poland was one of the countries that maintained the form of remote education as the basic form of work for the longest time (with exceptions, *e.g.*, regarding special institutions, including rehabilitation). One of the fundamental issues related to crisis remote education during the pandemic was access to tools enabling the conduct and participation in distance learning—both for students and teachers.

The nature of teachers’ profession indicates the need for the teacher to have appropriate personality traits and high mental resilience. Taking into account the different age of teachers and their personal predispositions, for some of them schoolwork may be a heavy burden and will not always be able to meet the requirements of the profession (Makowiec-Dąbrowska et al., 2021). The specificity of the teachers’ work predisposes to the occurrence of severe and chronic fatigue and professional burnout in this occupational group (Springer & Oleksa, 2017; Bortkiewicz et al., 2020; Khezerlou, 2013). In a study conducted in Japan among teachers, it was shown that the chronic fatigue syndrome found among the surveyed teachers was significantly higher than in the population of other working people across the country (Shimizu et al., 2011). The level of job satisfaction, which among teachers is related to their belief in a special role and professional mission, has a great impact on proper functioning (Bajcar et al., 2011). According to the analyzed studies, teachers with a low sense of job satisfaction are not satisfied with their personal development and their relations with the environment are dominated by a critical attitude causing uncertainty in everyday work and difficulties in establishing relationships. On the other hand, teachers with high job satisfaction have a positive self-image and can make a good impression on the environment. They are characterized by a great sense of responsibility, persistence, and consistency in everyday work. In relations with others, they are open and ready to provide help and support (Jakimiuk, 2018). Another important factor in the work of every teacher is his or her belief in self-efficacy, which is defined as the belief that they have sufficient opportunities to influence the achievements of their students. Many studies on teacher self-efficacy have shown that self-efficacy in teachers makes it much easier to cope with difficult situations and stress, which has a significant impact on the well-being and health of this professional group. It has been shown that beliefs about self-efficacy are negatively associated with mental tension, burnout, and depression, and positively with pro-health behaviors (Baka, 2017; Juczyński, 2001).

Teachers’ work during the COVID-19 pandemic was a great challenge for teachers and required them to abandon conventional teaching methods. It was related to spend more time, work, and creativity. When schools in Poland were closed (11/03/2020) and teaching throughout the country was carried out remotely, many teachers needed technical support, especially teachers from older generation (Tomczyk & Walker, 2021).

At the time, when we conducted our study (from May 24 to June 24, 2021) according to the Regulation of the Minister of Education, teaching in Poland was already conducted in direct contact with all the safety rules recommended by chief of sanitary inspector *i.e.,* using masks, social distance and hand disinfection. The exceptions were classes in which the COVID-19 virus infections were diagnosed, then pupils and teachers who had contact with the infected person were quarantined, and the learning was hybrid (*i.e.,* some in direct contact, some with the use of distance learning methods) (MEN, 2021; GIS, 2020).

Even though teaching returned to schools at that time, it was a difficult situation requiring the maintenance of the sanitary regime and the adaptation of children and adolescents to the related requirements. Not only some teaching topics needed to be recovered, but also the functioning of children and adolescents in a social group and switching back to learning in face-to-face contact. All these circumstances, together with the continued risk of COVID -19 infection, could affect job satisfaction, self-efficacy, and burnout in the studied groups of teachers (MEN, 2021; GIS, 2020). Teaching time in COVID-19 pandemic and between lockdowns was a completely new, global change in the way of teaching, which had never taken place in Polish education before. Therefore, it is not surprising that many schools lacked adequate equipment and guidelines regarding the rules of distance learning (Morgan, 2020; Romaniuk, Łukasiewicz-Wieleba & Kohut, 2020; Madalińska-Michalak, 2021; Rotas & Cahapay, 2020). A broader view of the functioning of teachers at that time presented Supreme Audit Office (SAO) report. A questionnaire survey conducted by the SAO in over five thousand educational institutions showed that that in the initial period of distance learning, teachers prepared for new conditions on their own, using the help of their younger colleagues, in March 2020 the number of teachers who completed various forms of education related to distance learning was 47%, and six months later it reached 81%. Most of the teachers working remotely used their own electronic equipment and the Internet, and only 7% of them used school resources. Nearly 70% of the teachers who participated in the SAO questionnaire conducted remote classes from home, and only 28% of them had access to a well-equipped classroom (Supreme Audit Office Report, 2021). The hardware deficiencies of the teachers themselves were the main problem for 10%, while for 46% they were the problem they dealt with (Buchner, Majchrzak & Wierzbicka, 2020). The specificity of the teachers’ work and extraordinary circumstances resulting from the pandemic were the basis for undertaking research on the key factors determining the appropriate health condition of this professional group and the level of education of children and adolescents closely related to it. After analyzing the most relevant findings of previous studies on job satisfaction, self-efficacy, and the level of professional burnout in teachers and its relationship with different contextual and personal variables, the main objectives and hypotheses of this study are discussed below.

To the authors’ knowledge, no research has yet assessed the level of occupational burnout, the sense of job satisfaction and the self-efficacy of teachers in the last pandemic in Poland. Therefore, the aims of this study were three-fold: (1) assess the level of occupational burnout, (2) the sense of job satisfaction and (3) the self-efficacy of teachers from primary and secondary schools working during the COVID-19 pandemic in Poland.

The following research problems were established:

- What is the level of job satisfaction, self-assessment of self-efficacy and the level of professional burnout in the surveyed teachers during the COVID-19 pandemic?
- Does the level of job satisfaction affect the self-efficacy level of professional burnout among the surveyed teachers?
- Do socio-demographic factors (*e.g.*, age, gender, job profile, etc.) differentiate the level of job satisfaction, self-efficacy and the level of professional burnout of the surveyed teachers?

## Materials & Methods

### Participants

The cross-sectional, descriptive study was conducted from May 25 to June 24, 2021, among 412 primary and secondary teachers from randomly selected (convenience sampling method) schools in the Podkarpacie region in Poland. We send invitations to 52 schools randomly selected *via* a randomized algorithm program. The sample size was determined with the help of the EPI INFO (StatCalc) software. 35 of the invited school gave positive feedback. Finally, the study group included teachers working in primary school and secondary school. The teachers participating in the study constituted a representative group for all teachers working in this region (the error threshold was 5%, *i.e.,* the test power was 0.95).

The method used was a diagnostic survey conducted by means of a questionnaire survey. Inclusion criteria: professionally active primary and secondary school teachers, minimum 2-year work experience, consent to participate in the study. The questionnaire contained socio-demographic data of the respondents and three standardized scales. A questionnaire containing all three scales (The Scale of Job Satisfaction (SJS), Generalized Self—Efficacy Scale (GSES) and AVEM (ger. Arbeitsbesorgenes Verhaltens und Erlebenmuster)) was provided to respondents in paper version. The teachers working at the school agreed to participate in the study were fully informed in writing and verbally about the nature of the study. They were assured of the voluntary participation in the survey and the anonymity of the answers provided. A questionnaire containing all three scales (SJS, GSES and AVEM) with the consent form was provided to the respondents in paper form. Each questionnaire had its own individual number and envelope to ensure the confidentiality and anonymity of the respondents. We distributed 1020 questionnaires and about 44% were collected back. Only the data from fully completed questionnaires (412) was used for the analysis. Data on types of work-related behavior (AVEM scale) were calculated using the software (UPSAmini), generating a table of results and a finished profile of the test subject. The SJS and GSES scores and ready-made types of work-related behavior were subjected to statistical analysis.

All participants were informed about the possibility of withdrawing from the study at any stage without any consequences.

### Tools

Self-efficacy was measured with the GSES scale (General Self-Efficacy Scale; Schwarzer, Jerusalem, in Polish adaptation of Juczyński) (Juczyński, 2001). The scale consists of 10 items and is designed to measure the general belief of an individual as to the feeling of effectiveness in specific situations, also related to the performed work. The answers are given on a 4-point scale (from 1—no/untrue, to 4—yes/completely true). The Polish version of the scale is characterized by good psychometric properties. The reliability of the scale is *α* = 0.88. The sum of all points gives the overall index self-esteem effectiveness, which can be between 10 and 40 points. The higher the score, the greater the sense of self-efficacy (Juczyński, 2001).

The results within 1–4 sten were considered low, and 7–10 as high, which corresponds to an area of about 33%, the lowest results and the same number of the highest scores in the scale. Results between 5 and 6 are considered average (Juczyński, 2001).

When analyzing the evaluation of teachers’ self-efficacy, the division of self-efficacy into two groups was used (only three percent of people with self-efficacy measured in sten < 5), resulting in a binary variable (low sten efficacy <7, high sten efficacy ≥ 7).

The Satisfaction with Job Scale allows to measure the cognitive aspect of overall job satisfaction. The scale includes five items rated on a seven-point scale:

- q1. In many respects my work is close to the ideal;

- q2. I have great working conditions;

- q3. I am satisfied with the work;

- q4. So far, I was able to achieve what I wanted, at work;

- q5. If I had to decide again, I would choose the same job.

Possible answers: from1—I strongly disagree to 7—I strongly agree.

The obtained results are summed up, and the overall score indicates the degree of satisfaction from work. The range of results is between 5 and 35 points (Zalewska, 2003).

The higher the score, the greater the sense of job satisfaction. The internal reliability of the scale is high, Cronbach’s alpha is 0.864. The reliability of the scale is *α* = 0.814. The Job Satisfaction Scale has been adapted to Polish conditions (Zalewska, 2003).

The analysis took into account teachers’ answers to individual questions contained in the job satisfaction scale. Using the clustering analysis (*k*-means method), three groups of teachers characterized by similar satisfaction were created.

The AVEM (ger. Arbeitsbesorgenes Verhaltens und Erlebenmuster) scale (authorship by Prof. Uwe Scharschmidt, Dr. Andreas W. Fischer, Polish adaptation conducted by Prof. Tatiana Rongińska, Prof. Dr. Werner Gaida), defines individual resources of an individual in the context of coping with the demands of professional situations (Rongińska & Werner, 2012). Importance is attached to explaining the ways of behavior and subjective assessment of interpersonal relations in the work environment. In practical terms, the use of the tool allows for the determination of behavioral patterns that are conducive to the mental health of an individual and a positive attitude to work. AVEM makes it possible to identify patterns of behavior and experiences that pose a threat to the health of an individual. They are considered depending on the relationship in the work environment and the immediate environment of the individual. The practical application of the method consists of developing pro-health preventive actions. The questionnaire consists of 66 items (Rongińska & Werner, 2012). The examined person assesses the accuracy of each of the statements in relation to their own feelings, experiences, and experiences on a five-point scale. The area of behavior and experiences in task situations is described by 11 scales of the questionnaire (each of the scales corresponds to 6 tasks-statements). Reliability tested by Cronbach’s alpha method for individual scales ranges between 0.78 and 0.87. Determining the reliability with the split half method (according to Spearman-Brown) gave a result between 0.76 and 0.90. The stability coefficients obtained so far (over a period of 3 months) for the German version are between 0.69 and 0.82. The examination takes approximately 10 min. A computer program is used for the test, which ensures the completeness of the answer. The analysis of individual results is based on comparing the values of the raw scales calculated according to the key attached to the test with the norms of the selected sample, plotting the profile and comparing it with four reference profiles corresponding to a specific type of behavior and experience. The AVEM evaluation program automatically calculates the values of all the scales provided for in the test and compares them to the norms of the sample selected by the user. It generates a table with results and plots the profile of a given person together with reference profiles and the probability of belonging to a specific pattern-type of behavior and experiences.

There are four fixed types of work-related behavior and experiences:

**Type G**—healthy type

Committed, distancing, balanced, prone to offensive problem-solving strategies, the person is an example of a positive attitude to work reinforced by the mobilizing influence of emotions.

**Type S**—savings type

About average professional ambitions, a reduced level of motivation, a clear tendency to distance from work-related problems, satisfied with the results of his work, with a positive attitude to life. The person is characterized by a low subjective meaning of work, low professional ambitions, and a lack of perfectionism.

**Type A**—overburdened risk type

Ascribing to work a very high subjective importance, with low mental resistance and high intensity of negative emotions at the same time

**Type B**—burnout type

It is characterized by a very low subjective meaning of work, reduced resistance to stress with a simultaneous limited ability to distance oneself, a tendency to quit in difficult situations and extremely low internal balance values (Rongińska & Werner, 2012).

The authors have permission to use mentioned scales from the copyright holders.

### Statistical analysis

The estimation method and the following statistical methods were used: in order to present the data, the method of descriptive statistics was used—arithmetic mean (M), =and standard deviation (SD).

The UPSAmini software, license agreement number: UR/20150706/EDU/2, was used to calculate the AVEM questionnaire and identify the types of work-related behavior.

The Shapiro–Wilk test was used to verify the data distribution.

The median with an interquartile range and the mean with standard deviation were used to present continuous variables. Mann–Whitney *U* test or unpaired *t*-test, ANOVA or Kruskal–Wallis’s test were used to compare these variables. Categorical variables were given as percentages and compared with the *χ*^2^ test. Spearman’s rank correlation test was used to assess the relationship between the variables. For cluster analysis *k*-means clustering method was used. We obtained three different clusters of satisfactions. Three remaining clusters were compared by the covariance analysis (ANOVA), Kruskal–Walli’s test or *χ*^2^ test, as appropriate. We calculate for binary variable the odds ratio (OR) with 95% confidence interval (CI). In order to calculate OR, if, apart from the binary variable, the second variable was a quantitative (numerical) variable, then the ROC analysis was first used to obtain a significant cut-off point, and then this numerical variable was changed to a binary variable gr1 -all elements < cut point and group 2—all elements ≥ cut point and a pattern is applied using the Statistica TIBCO 13.3 software.

To create a model describing effectiveness, we used the discriminant tree method.

Used not only simple and commonly used tests, but also the Clustering Analysis and Discriminant Tree Methods (DTM, which are not statistical tests), which allow to indicate the interaction of two features to each other and to build groups (clusters) of people similar to each other in terms of many selected features and on the basis of their responses. After that the simple tests allowed for an analysis that showed other features significantly differentiating the constructed clusters.

Thus, DTM analysis allows to illustrate which features should be considered, in what order and in terms of their values to be able to distinguish (predict) the level of the feature under study.

We used this method to illustrate that it is possible to predict the value of the tested feature based on a much smaller number of questions, what is more, this method shows what we should ask about to predict the value of the tested feature and how to classify the questions.

The statistical significance was set at *p* < 0.05.

The Statistica TIBCO 13.3 and R (version 3.6.1) software were used for statistical analysis.

### Ethics

This research project was carried out in accordance with the Helsinki Declaration. The study was approved by the institutional Bioethics Committee at the University of Rzeszów (Resolution No. 13/05/2021) and all appropriate administrative bodies.

## Results

### Characteristics of the study group

A total of 412 teachers working in primary (*N* = 115) and secondary (*N* = 298) schools participated in the study. More than half of the respondents were women (*N* = 270; 65.38%), men accounted for 34.38% of participants (*N* = 142). The average age of the respondents was 41.7 years, and the average work experience of the surveyed teachers was 17.02 years. The vast majority (*N* = 314; 76.03%) had the title of certified teacher and 118 (28.57%) was the class tutor. Self-efficacy assessed using the GSES scale in most of the respondents was at a high level (high sten ≥ 7) and the level of job satisfaction at a medium level (21.92). Almost half of the respondents (*N* = 200; 48.4%) presented type B of work-related behavior (Table 1).

**Table 1:** Characteristics of the study group.

**Independent variables**	**Categories**		***N***	**%**	**Mean (95%CI)**	**SD**	**Median (q1–q3)**
Sex	Female		270	65.38			
	Male		142	34.38			
Age	All together				41.7(40.89;42.51)	8.36	40(37;47)
Age divided into age groups	Up to 37 years		111	26.88			
	From 38–47 years old		200	48.43			
	Over 48 years		102	24.70			
Place of work	Secondary school		298	72.15			
	Primary school		115	27.85			
Work experience	All together				17.02(16.19;17.84)	8.57	17(12;23)
Work experience divided into age ranges	Up to 5 years		58	14.04			
	From 5–15 years		103	24.94			
	From 15–24 years		170	41.16			
	Over 24 years		82	19.85			
Held position	Certified teacher		314	76.03			
	Contract teacher		40	9.69			
	Trainee teacher		23	5.57			
	Appointed teacher		35	8.47			
Class tutor	Yes		118	28.57			
	No		291	70.46			
Self-assessment of efficacy (GSES)	Low-medium (sten < 7)		107	25.91			
	High (sten > = 7)		306	74.09			
	Efficacy [in sten]				7.36(7.2;7.52)	1.63	7(6;8)
	Efficacy [in points]				31.93(31.52;32.34)	4.26	31(29;35)
Type of work-related behavior (AVEM)	Type B		200	48.42615			
	Type G		48	11.62228			
	Type S		32	7.74818			
	Type A		133	32.20339			
Level of job satisfaction (SJS)	Satisfaction sum				21.92(21.37;22.46)	5.61	23(18;26)
	Satisfaction %				62.62(61.07;64.16)	16.02	65.71 (51.43;71.43)
	Satisfaction after clustering	Group 1	83	20.10			
		Group 2	141	34.14			
		Group 3	189	45.76			

**Notes.**

SD standard deviation GSES General Self-Efficacy Scale AVEM ger. Arbeitsbesorgenes Verhaltens und Erlebenmuster Type B, G, S, A type of work-related behavior SJS The Scale of Job Satisfaction Group 1, 2, 3 the teachers divided into groups (1–3) presenting the appropriate level of job satisfaction

#### Level of job satisfaction

The third group consists of teachers with the highest level of job satisfaction in all areas of the scale (for all 5 questions), obtaining statistically significantly higher points than teachers in group 2 and group 1. For all comparisons of group 3 with groups 1 and 2 *p* < 0.0001. Group 2 is the group with average satisfaction for questions 1, 3, 4, 5 statistically significantly higher values (for all *p* < 0.0001) for question 2, there are no significant differences between groups 2 and 1 (*p* = 0.08). Multiple comparison tests with appropriate corrections were used to compare the groups. Additionally, analyzing which questions statistically significantly influencing the establishment of satisfaction clusters using the analysis of variance, it was obtained that the answers to all of the five questions mentioned had a statistically significant influence on the division (*p* < 0.0001 in each case).

#### Job satisfaction and self-efficacy assessment

Considering the results concerning job satisfaction and self-efficacy assessment of the surveyed teachers, it was shown that job satisfaction had a statistically significant impact on the assessment of self-efficacy of the surveyed teachers (*p* < 0.0001). In the group of teachers with a high level of job satisfaction, a high self-efficacy assessment is 2.5 times more frequent and statistically significant than a low self-efficacy assessment. In the first and second groups, *i.e.,* among teachers with medium and low job satisfaction, this ratio is also statistically significant, but lower than 1 (*i.e.,* statistically significant, low self-efficacy prevails over high. The ratio of high self-efficacy to low ones was obtained by performing the analysis the odds ratio from the Ratio Table 2).

**Table 2:** The correlation of self-efficacy assessment on job satisfaction among the surveyed teachers, analysis of the odds ratio against the ratio.

**Level of job satisfaction**	***p***	**Od ratio**	**95% CI lower**	**95% CI upper**
		**High self-efficacy rating/ Low self-efficacy rating**		
Low	0.0016	0.572	0.404	0.810
Average	0.0202	0.697	0.514	0.945
Hight	0.0000	**2.511**	1.716	3.676

**Notes.**

*p*-*p*-value, indicate significant values (*p* < 0.05).

CI confidence interval

The types of work-related behavior have a statistically significant impact on the assessment of the teachers’ self-efficacy. Carrying out the analysis for the selected types of work-related behavior (*p* < 0.0001), it was shown that in the group of teachers presenting the G behavior type, high self-efficacy scores are 3,272 times more frequent than low self-efficacy scores. In the group of teachers presenting the behavior type B and S, low self-efficacy assessment is more frequent (*p* < 0.05). (Table 3).

**Table 3:** Type of work-related behavior and self-efficacy assessment.

**Types of work-related behavior (AVEM)**	***p***	**Od ratio**	**95% CI lower**	**95% CI upper**
		**High self-efficacy rating/ Low self-efficacy rating**		
Type B	0.0128	0.604	0.406	0.898
Type G	0.0036	3.272	1.472	7.273
Type S	0.0003	0.337	0.186	0.612
Type A	0.0888	1.501	0.940	2.396

**Notes.**

*p*-*p*-value, indicate significant values (*p* < 0.05).

CI confidence interval

The level of job satisfaction among the surveyed teachers, measured by the number of points, also statistically significantly differentiates the group of teachers with low self-efficacy scores from teachers with high self-efficacy scores; *p* < 0.0001 as a higher efficacy stimulant. By analyzing and dividing the level of job satisfaction into three groups, other factors influencing the level of job satisfaction among teachers were revealed: age by groups (*p* < 0.0001), seniority work broken down into groups (*p* = 0.0003), workplace (*p* = 0.008), position (*p* = 0.0001) and type of behavior (*p* < 0.0001).

Moreover, a statistically significant influence of the age of the surveyed teachers (*p* = 0.01) and seniority in years (0.004) is visible. Analyzes of multiple comparisons with appropriate corrections show that in cluster 2 there are statistically significantly older teachers than in cluster 3 (*p* = 0.0122), and in cluster 2 there are people with longer work experience than in cluster 3 (*p* = 0.0049) (Table 4).

**Table 4:** Level of job satisfaction depending on selected factors (age, work experience, workplace, position held, type of work-related behavior).

**Percentage distribution of the chances of belonging to a given satisfaction cluster, if one is in one age group**			
**Age—division into age groups**	**Level of Job satisfaction—clusters devision**		
	**1 (%)**	**2 (%)**	**3 (%)**
Up to 37 years	18.02%	14.41%	67.57%
From 38–47 years old	23.00%	42.00%	35.00%
Over 48 years	16.67%	40.20%	43.14%
**The percentage distribution of the chances of belonging to a given satisfaction cluster on the condition that one is in a given group of work experience**			
**Work experience—age group**	**Level of Job satisfaction—clusters division**		
	**1 (%)**	**2 (%)**	**3 (%)**
Up to 5 years	12.07%	13.79%	74.14%
From 5–15 years	24.27%	30.10%	45.63%
From 15–24 years	20.59%	44.71%	34.71%
Over 24 years	19.51%	31.71%	48.78%
**The percentage distribution of the chances of belonging to a given satisfaction cluster on the condition that one is in a given workplace**			
**Place of work**	**Level of Job satisfaction—clusters division**		
	**1 (%)**	**2 (%)**	**3 (%)**
Secondary school	20.81%	37.92%	41.28%
Primary school	18.26%	24.35%	57.39%
**The percentage distribution of the chances of belonging to a given satisfaction cluster depending on the type of work-related behavior**			
**Types of work-related behavior**	**Level of Job satisfaction—clusters division**		
	**1 (%)**	**2 (%)**	**3 (%)**
Type B	24.00%	41.50%	34.50%
Type G	4.17%	14.58%	81.25%
Type S	21.88%	50.00%	28.13%
Type A	19.55%	26.32%	54.14%

The analysis of the results showed that the type of work-related behavior among the surveyed teachers depends on age by group (*p* = 0.016), seniority by group (*p* = 0.016), and position (*p* < 0.0001) (Table 5).

**Table 5:** The type of work-related behavior depending on selected factors (age group, work experience and held position).

**Age—division into age groups**	**The type of work-related behavior in age groups**			
	**Type** **B (%)**	**Type** **G (%)**	**Type** **S (%)**	**Type** **A (%)**
Up to 37 years	37.84%	18.02%	4.50%	39.64%
From 38–47 years old	54.50%	8.00%	8.00%	29.50%
Over 48 years	48.04%	11.76%	10.78%	29.41%
**Work experience—age rangesgroup**	**The chances of occurrence of the type of work-related behavior in work experience—age group**			
	**Type** **B (%)**	**Type** **G (%)**	**Type** **S (%)**	**Type** **A (%)**
Up to 5 years	32.76%	27.59%	6.90%	32.76%
From 5–15 years	61.17%	6.80%	4.85%	27.18%
From 15-24 years	47.06%	10.00%	9.41%	33.53%
Over 24 years	46.34%	9.76%	8.54%	35.37%
**Held position**	**The type of work-related behavior depending on held position**			
	**Type** **B (%)**	**Type** **G (%)**	**Type** **S (%)**	**Type** **A (%)**
Certified teacher	51.59%	7.64%	7.01%	33.76%
Contract teacher	30.43%	13.04%	4.35%	52.17%
Trainee teacher	48.57%	5.71%	14.29%	31.43%
Appointed teacher	32.50%	47.50%	10.00%	10.00%

**Figure 1:**
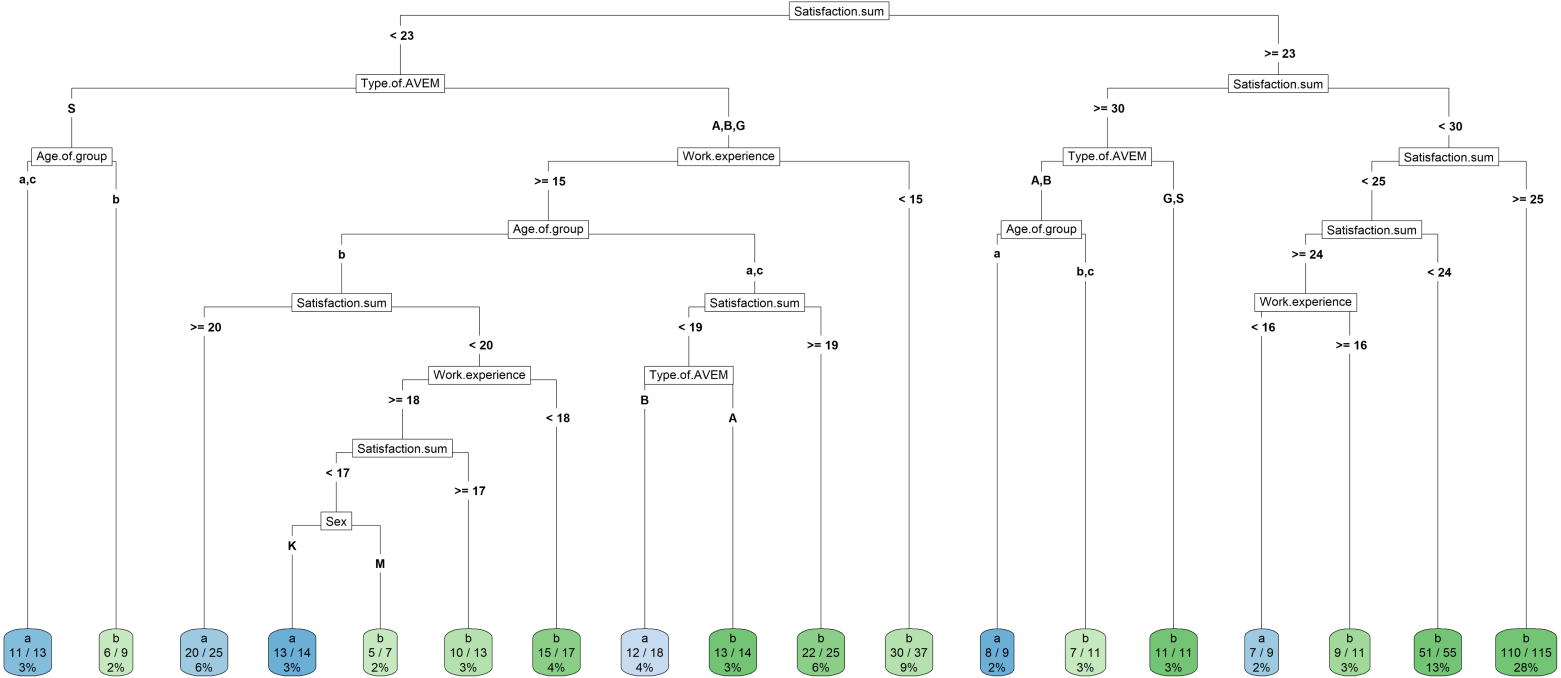
Discriminant tree analysis. Group “a” blue—low-medium effectiveness, —group “*b*” green—high effectiveness, type B, G, S, A—type of work-related behavior. The darker the color, the better the path in the tree differentiates this group.

The discriminant tree analysis showed that, considering factors such as sex, age, job satisfaction, types of work-related behavior and work experience, it is possible to assess the teacher’s self-efficacy assessment. The tree shows, step by step, how to determine whether a given teacher will achieve low-medium effectiveness—group “*a*” or high effectiveness—group “*b*”. The long branches of the tree describe which of the self-efficacy groups a given teacher belongs to, while the lower leaves contain the self-efficacy groups, the chance of belonging to a given group and the percentage of people from all those in the given tree branch (Fig. 1).

**Figure 2:**
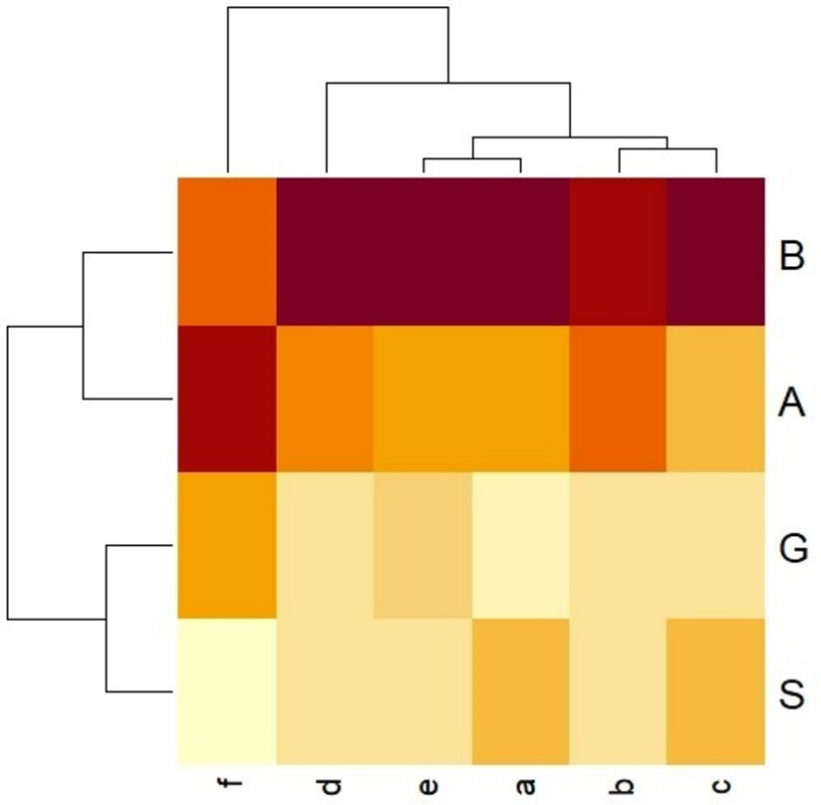
Heat map: Interrelationships between teachers’ job satisfaction and the assessment of self-efficacy and the type of work-related behavior. Type B, G, S, A—type of work-related behavior. a, b, c, d, e, f,—combined groups of satisfaction and efficacy (a, low satisfaction, low efficacy; b, low satisfaction, high efficacy; c, average satisfaction, low efficacy; d, average satisfaction, high efficacy; e, high satisfaction, low efficacy; f, high satisfaction, high efficacy). The darker the colors, the greater the relationship.

The mutual dependencies between the satisfaction with teachers’ work and the assessment of their self-efficacy and the type of work-related behavior are illustrated by the “heat map”. The darker the color of the field, the stronger the impact and the connecting lines show clusters of close answers in one question (Fig. 2).

## Discussion

The aim of the study was to assess the level of occupational burnout, the level of job satisfaction and the self-efficacy of teachers working during the COVID-19 pandemic in Poland. The issue of teacher satisfaction with professional work takes a special place both in the field of pedagogical research and in the social dimension. Positive results of the teacher’s work and his commitment are related to the feeling of professional satisfaction. In the social dimension, the feelings associated with the work of teachers are of greater importance than in the case of people working in other professions (Buchcic, 2014). Our results may show that the satisfaction with the work of the surveyed teachers was average. Satisfaction with work, also known as job satisfaction, is a subjective state, but there is a common belief that the teacher’s work is hard, and stress and fatigue are a common phenomenon, leading to burnout (Scheuch, Haufe & Seibt, 2015; Mukundan & Ahour, 2011; Yu et al., 2015).

Even before the COVID-19 pandemic, teachers’ job satisfaction differed from country to country. Zieger, Sims & Jerrim (2019) conducted a large-scale study using TALIS (Teaching and Learning International Survey) data (OECD, 2013). The researchers found that secondary school teachers in England had lower job satisfaction as a teacher compared to the other 17 other countries, including Poland (Zieger, Sims & Jerrim, 2019). On the other hand, the study conducted by Zakariya, Bjørkestøl & Nilsen (2020) on teacher’ job satisfaction from 38 countries showed that Austria, Chile, Spain, Canada, and Argentina are the countries where teachers have the highest levels of job satisfaction, while the least satisfied with their jobs were teachers in Bulgaria, England, Portugal, Saudi Arabia and Malta. The time of the COVID-19 pandemic was a great challenge for many teachers and significantly reduced their job satisfaction (Muhammad et al., 2021; Dicke et al., 2020). The results of our study can indicate how difficult the pandemic was for many teachers. The vast majority of the surveyed teachers (200 people) presented type B of work-related behavior, *i.e.,* burnout, and 133 people presented type A, *i.e.,* the type of personality in which the probability of burnout is very high. Thus, 333 teachers out of 410 participating in the survey did not cope with a difficult situation related to their work. Similar results presented Karbanowicz, in her study, 90% teachers also presented type B, *i.e.,* burnout (Karabanowicz, 2014). Increasing demands and the necessity to cross conventional teaching methods during the national lockdown mean that more and more teachers show symptoms of increasing fatigue due to unfavorable working conditions. This is, of course, an individual situation and depends on the intensity of stressors as well as the subjective sensitivity and mental resilience of a given person, but it shows that many teachers have a problem with coping with the difficulties they experience in their daily work (Buchner, Majchrzak & Wierzbicka, 2020; Muhammad et al., 2021; Dicke et al., 2020). In our study, the vast majority (*N* = 306) showed a high level of self-efficacy, which proves that the surveyed teachers are convinced that their own ability to plan, organize and conduct the teaching process in an effective manner, conducive to achieving the assumed educational goals. The issue of self-efficacy among teachers is the subject of many studies (Skaalvik & Skaalvik, 2007; Tschannen-Moran & Woolfolk, 2001). It has been noticed that teachers with a high sense of self-efficacy introduce modern teaching methods more often than teachers with a low sense of self-efficacy, which significantly improves not only the effectiveness of teaching, but also increases the level of job satisfaction (Dilekli & Tezci, 2016; Barouch, Adesope & Schroeder, 2014).

Considering the results concerning job satisfaction and self-efficacy assessment of the surveyed teachers, it was shown that job satisfaction may have a statistically significant impact on the assessment of self-effectiveness of the surveyed teachers. Raily et al. points to similar dependencies, that high self-efficacy is an important determinant of job satisfaction among Irish teachers (Reilly, Dhingra & Boduszek, 2014). Considering the COVID-19 pandemic and the new challenges for teachers related to it, their sense of self-efficacy can be an important factor determining the entire area of the teacher’s work, taking into account the sense of job satisfaction and the risk of developing professional burnout (Pressley, 2021a; Hoang et al., 2020; Pressley, 2021b). The review of available publications shows that teachers during COVID-19 showed significantly lower self-efficacy. This is confirmed by the results of the American study in this area. The authors indicate that both virtual and hybrid teachers had a lower sense of self-efficacy compared to teachers teaching in direct contact. In addition, researchers indicate that an important factor influencing the sense of effectiveness is the level of qualifications (Pressley & Ha, 2021).

The conducted research showed that age and seniority were factors that significantly may influence the level of satisfaction with the work of the surveyed teachers. It is consisted of Lisowska study, that younger teachers and teachers with work experience from 1 to 5 years show a higher level of job satisfaction compared to older people working from 6 to 20 years (Lisowska, 2017). In turn, in Shresth’s study, teachers of older age groups expressed greater satisfaction with their work than their younger colleagues (Shrestha, 2019). According to Okpara, Squillace & Erondu (2005) the respondents’ gender is also a factor influencing the level of teachers’ job satisfaction. Females presented a higher level of job satisfaction with compared to males. The analysis of the results showed that the type of work-related behavior among the surveyed teachers depends on age, seniority and held position. It is in opposite to Karabanowicz (2014), where the seniority and held position did not determine the type of work-related behavior among the surveyed teachers. The results of Smetackova study conducted among 2,394 Czech teachers showed negative correlation between burnout and self-efficacy. Teachers who scored high in the self-efficacy reported low burnout symptoms, and vice versa, and as in our study, the risk of burnout was higher among older teachers and with 6 to 20 years of work experience (Smetackova, 2017), it is consistent with Skaalvik & Skaalvik (2007).

The research shows the scale of the problem of professional burnout the research can be used by headmasters, decision makers, and local governments to show the magnitude of the teachers’ problem and possibly provide adequate psychological support for teachers

### Limitations and future research

To our knowledge, it is a one of the first study undertaking teachers’ work during the COVID-19 pandemic in Poland one of the few in the world. The research uses standardized scales and advanced statistical methods, which significantly increases the research value of the article. The conducted study highlights important relationships between the level of self-efficacy, coping with workload, professional burnout, and the job satisfaction among the surveyed teachers. Our study has some limitations that should be considered when analyzing the results. The time of the study is a very difficult period of teachers’ functioning related to the pandemic and the results should be analyzed in this context. To reassess the level of job satisfaction, sense of effectiveness and burnout level of Polish teachers a repetition of the study after the pandemic period is being considered. The study was conducted in one of the regions of the country and should be repeated on a larger population among other parts of Poland. Being that the study is cross-sectional, the causality and temporality issues should not be considered.

## Conclusions

The article is in line of other studies analyzing the functioning teachers during a COVID-19 pandemic. These circumstances influenced teachers’ level of effectiveness, job satisfaction and predispose them to the occurrence of burnout syndrome. The obtained results show that assessment of self-efficacy in most of the respondents was at a high level and the level of job satisfaction at a medium level. Almost half of the respondents presented burnout type (B) of work-related behavior. Self-efficacy was influenced by the level of work satisfaction and the type of work-related behavior. Variables such as age and seniority influenced the level of job satisfaction, while the type of behavior related to work was influenced by age, seniority, and the held position.

## Supplemental Information

10.7717/peerj.13349/supp-1 Data S1 Raw data

## Additional Information and Declarations

### Data Availability

The following information was supplied regarding data availability: The raw measurements are available in the Supplementary File.
